# Emergence and expansion of highly infectious spike protein D614G mutant SARS-CoV-2 in central India

**DOI:** 10.1038/s41598-021-95822-w

**Published:** 2021-09-13

**Authors:** Shashi Sharma, Paban Kumar Dash, Sushil Kumar Sharma, Ambuj Srivastava, Jyoti S. Kumar, B. S. Karothia, K. T. Chelvam, Sandeep Singh, Abhaydeep Gupta, Ram Govind Yadav, Ruchi Yadav, T. S. Greeshma, Pramod Kumar Kushwaha, Ravi Bhushan Kumar, D. P. Nagar, Manvendra Nandan, Subodh Kumar, Duraipandian Thavaselvam, Devendra Kumar Dubey

**Affiliations:** 1 Virology Division, Defence Research and Development Establishment, Gwalior, Madhya Pradesh 474002 India; 2 COVID-19 Diagnosis Task Force, Defence Research and Development Establishment, Gwalior, Madhya Pradesh 474002 India

**Keywords:** SARS-CoV-2, Molecular evolution

## Abstract

COVID-19 has emerged as global pandemic with largest damage to the public health, economy and human psyche.The genome sequence data obtained during the ongoing pandemic are valuable to understand the virus evolutionary patterns and spread across the globe. Increased availability of genome information of circulating SARS-CoV-2 strains in India will enable the scientific community to understand the emergence of new variants and their impact on human health. The first case of COVID-19 was detected in Chambal region of Madhya Pradesh state in mid of March 2020 followed by multiple introduction events and expansion of cases within next three months. More than 5000 COVID-19 suspected samples referred to Defence Research and Development Establishment, Gwalior, Madhya Pradesh were analyzed during the nation -wide lockdown and unlock period. A total of 136 cases were found positive over a span of three months that included virus introduction to the region and its further spread. Whole genome sequences employing Oxford nanopore technology were generated for 26 SARS-CoV-2 circulating in 10 different districts in Madhya Pradesh state of India. This period witnessed index cases with multiple travel histories responsible for introduction of COVID-19 followed by remarkable expansion of virus. The genome wide substitutions including in important viral proteins were identified. The detailed phylogenetic analysis revealed the circulating SARS-CoV-2 clustered in multiple clades including A2a, A4 and B. The cluster-wise segregation was observed, suggesting multiple introduction links and subsequent evolution of virus in the region. This is the first comprehensive whole genome sequence analysis from central India, which revealed the emergence and evolution of SARS-CoV-2 during thenation-wide lockdown and unlock.

## Introduction

Emergence of novel virus is always considered as a major challenge to humanity. SARS-CoV-2, the etiological agent of “coronavirus disease 2019” (COVID-19) pandemic has been causing devastating damage to global public health and economy since its emergence in December 2019^1,2^. The common signs of infection include cough, fever, sore throat, respiratory symptoms inclusive of difficulties in breathing. More severe symptoms can include pneumonia, severe acute respiratory syndrome, kidney failure and even death with coalescence of factors^2–4^. Many COVID-19 cases have been reported to be asymptomatic and they serve as carrier of SARS-CoV-2^5^.

SARS-CoV-2, is a novel member of subgenus- *Sarbecovirus*, genus- *Betacoronavirus* of family- *Coronaviridae*. Whole genome sequences (WGS) of SARS-CoV-2 suggest RaTG13 bat-CoV to be its closest progenitor with 96% homology^6^. India took drastic steps to contain the spread of the virus including imposition of strict travel restrictions that led to a pause in new cases up to February 2020^7^. However, since March 2020 onwards, India also witnessed a surge of cases mostly in people coming from Europe, US, China and South East Asia, which was further complicated by local transmission. In March, impositions of nationwide lockdown successfully contained the epidemic curve. Despite these measurements, the exodus of individual from their place of work to native places during later part of lockdown led to rapid transmission of virus to remote corners of the country. Further lifting of lockdown and with early phases of Unlock, India witnessed explosive outbreaks, quickly occupying the 3^rd^ position in COVID-19 burden in the world on 6th July, 2020.

The genome of SARS-CoV-2 is a single stranded positive sense RNA of approximately 29.9 kb long. It encodes four structural proteins viz., Spike (S), Envelope (E), Membrane (M) and Nucleocapsid (N); fifteen non structural proteins (nsp1-nsp10 and nsp12-nsp16) and some accessory proteins (e.g., 3a, 3b, p6, 7a, 7b, 8b, 9b). Genetic characterization of a virus is extremely important from both epidemiological and microbial forensics points of view. We at Defence Research & Development Establishment (DRDE) Gwalior being a nodal center for COVID-19 diagnosis were engaged in screening of COVID-19 suspected cases referred from 10 different disricts of Madhya Pradesh. A total of 5010 samples were referred with detailed patient history from the district health authorities to DRDE, Gwalior for molecular diagnosis during mid of March to May 2020. Next generation sequencing (NGS), an emerging technology has aided in understanding the evolution of SARS-CoV-2 genomes and its transmission patterns after it enters a new population^8,9^. This technology has been proven to be an important tool towards formulating strategies for management of this pandemic. In this study, we characterized the genome of representative viruses involved in index cases of the region along with it’s expansion and fatal cases. Further, the whole genome analysis was performed to annotate genome wide amino acid substitutions. The detailed molecular phylogentic analysis was carried out to classify viruses into different clusters and to understand their transmission linkages focusing on the effect of nation-wide lock down and Unlock.

## Results

### Clinical representation and laboratory diagnosis of COVID-19 cases

**Figure 1:**
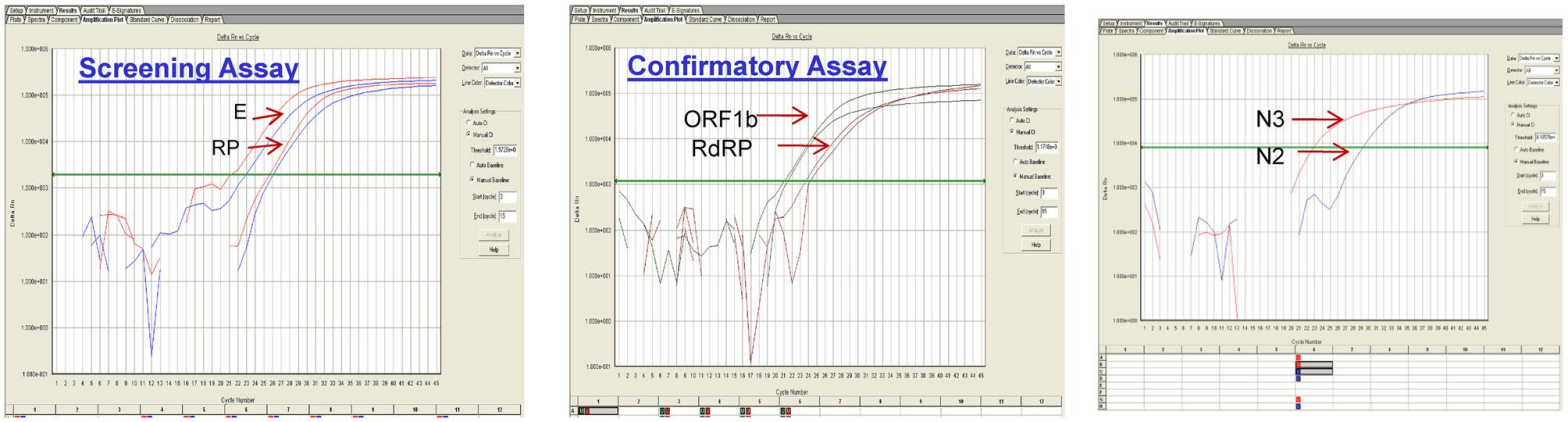
Representative amplification plot of SARS-CoV-2 positive samples showing amplification curve against different gene targets (E/RnaseP, Orf1b/RdRp, N genes).

One hundred thirty six cases out of 5010 samples (March–May 2020) referred to DRDE, Gwalior for laboratory diagnosis of COVID-19 were found positive. All samples were investigated by WHO approved real time RT-PCR using SARS-CoV-2 specific primers and probes for E, RNaseP, RdRP and N genes. RNaseP as a housekeeping control was included to ensure sample collection quality. All positive samples had shown clear positive amplification curve for presence of above mentioned genes (Fig. 1). The overall RT-PCR positivity was 4%. The minimum age of COVID-19 infected cases was 05 months whereas, oldest observed case was 98 years old. The maximum positive cases belonged to 21 to 30 years age group. Death in immuno-compromised patients was reported within 24–48 h of hospitalization. The clinical history revealed that the primary symptoms ranged from fever, sore throat and breathlessness. Maximum case positivity (68%) was reported from district Morena.

### Whole genome sequencing of SARS-CoV-2

**Figure 2:**
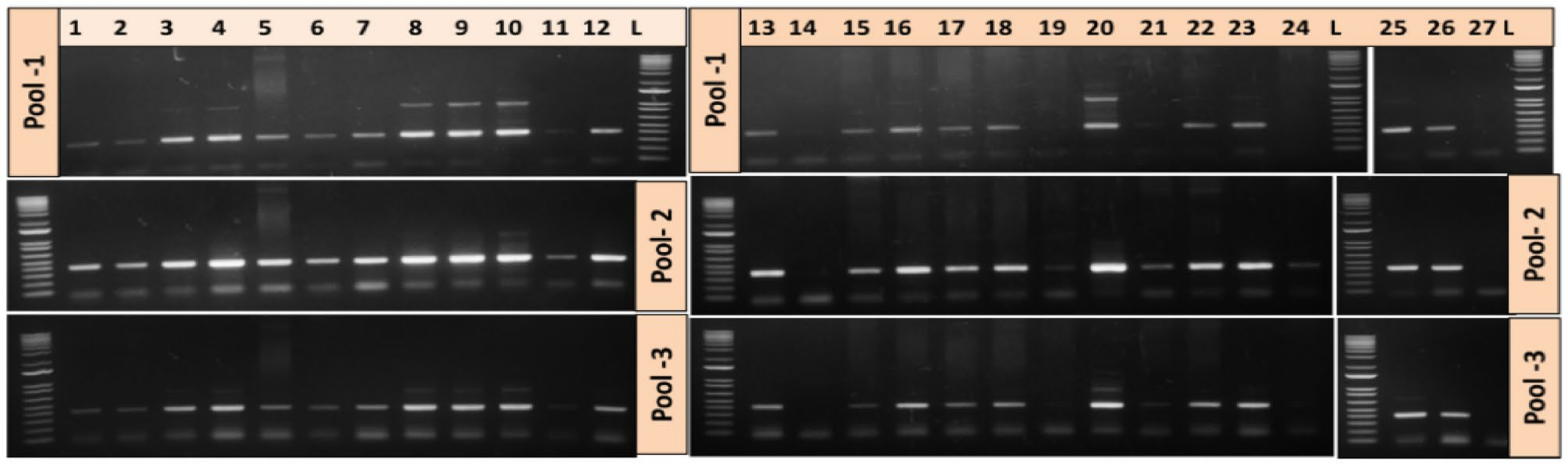
Agarose gel electrophoresis of various amplicons of representative positive samples generated using SARS-CoV-2 specific primer pools. These amplicons were further used for nanopore sequencing.

Twenty six representative samples as detailed in Supplementary Table 1 along with GISAID accession numbers, patient age, location were sequenced in this study. The Ct values of all these 26 samples in respect to different genes are provided in Table 1. The viral RNA from above samples were converted to cDNA and the same was processed for amplification through 3 pools of specific primers (Fig. 2). The NGS led to generation of around 0.03 to 0.30 million nanopore reads. The length of RNA genomes was found to be 29.89 kb. The sequencing coverage for all samples was in range of 590X to 5000X showing high depth of sequence data. The processed reads from Guppy was used for variant calling using Arctic pipeline. The good quality filtered variants obtained were in the range of 5–13 across 24 samples. The predicted variants were further annotated using SnpEff TOOL. The reference genome of SARS-CoV-2 was used for annotating good quality variants. The alignment files from Arctic pipeline were used for generating consensus sequence based on read depth of 10, supporting a position across the genome. The bases present in consensus sequence are of two types: A, G, C, T having read depth of 10 or more and lower case base a, t, g, c, n having read depth of less than 10 a/t/g/c and read depth of 0(n). Around 30 kb size consensus was generated for all samples with a range of 0.003–3.86% N’s (non-ATGC) observed in the consensus.

**Table 1:** Ct values of samples sequenced in this study with respect to different gene targets detected by COVID-19 Taqman qRT-PCR.

Sample no	RdRP (Ct value)	ORF 1 ab (Ct value)	N2 (Ct value)
1	27	24.9	23.8
2	26.2	23.2	25.3
3	23.8	21.2	21.3
4	27.9	26.2	27.7
5	30.6	28.6	29.1
6	23.2	22.3	21.4
7	29	28	29
8	28	27.7	28.2
9	29	28	28
10	27.2	26.1	26.7
11	26.5	24.1	25.2
12	20.5	18.4	18
13	26.2	25	23
14	29.7	27	26.1
15	29.1	27.1	28
16	29.8	28.9	29.2
17	30.9	29.6	30
18	28.1	25.5	25.2
19	18.8	29.2	29
20	31.3	27.9	27.5
21	26.5	26.5	22
22	21.5	19.3	17.5
23	33.2	30.9	29.6
24	29	26.2	27.1
25	33.2	31.3	32.5
26	23.8	24.1	23.5

### Molecular analysis of whole genome sequences

The SARS-CoV-2 genome sequences including viruses from different global locations and within India for a period of December 2019 to May 2020, were retrieved from GISAID, EpiCov global reference web interface representative cases (Supplementary Table 2). The amino acid substitutions were found scattered throughout the genome. A total of 38 amino acid substitutions were observed compared to prototype Wuhan strain. Out of these, 24 were found in ORF1ab, 5 in the S protein, 2 in ORF 3a, 1 each in E, M and ORF 7a. Finally, 4 substitutions were located in the N protein. The unique non-synonymous variants from all samples are shown in Table 2.

**Table 2:** Details of unique amino acid substitutions of SARS-CoV-2 sequenced in this study compared to prototype strain isolated from Wuhan (MN908947).

Amino acid position	Gene name	Prototype SARS-CoV-2 (MN908947)	Virus sequenced in this study
27	Orf1ab	L	F
453	Orf1ab	I	T
690	Orf1ab	A	V
1158	Orf1ab	P	S
1515	Orf1ab	S	F
1534	Orf1ab	S	I
1589	Orf1ab	T	I
1597	Orf1ab	T	I
1812	Orf1ab	A	D
2016	Orf1ab	T	K
2032	Orf1ab	D	Y
2586	Orf1ab	V	G
3652	Orf1ab	M	I
3811	Orf1ab	Y	D
4207	Orf1ab	T	I
4394	Orf1ab	A	V
4489	Orf1ab	A	V
4661	Orf1ab	D	N
4696	Orf1ab	D	G
4715	Orf1ab	L	P
4721	Orf1ab	L	I
5041	Orf1ab	S	L
6083	Orf1ab	P	S
6471	Orf1ab	Q	K
583	S	E	D
614	S	D	G
884	S	S	F
929	S	S	T
943	S	S	P
81	ORF 3a	C	R
155	ORF 3a	D	Y
68	E	S	F
214	M	S	I
104	ORF 7a	V	F
13	N	P	L
18	N	G	S
139	N	L	F
378	N	E	V

Important non-synonymous variants includes RdRp: A97V observed in 7 strains including virus introductory index cases from three different districts, N: P13L observed in 4 strains, NSP3: T2016K observed in 7 strains including introductory index cases from three different districts. Five amino acid substitutions were observed in spike protein viz., E583D in a strain from Datia, D614G observed in 17 strains from Morena, Datia, Gwalior, Ashoknagar, S884F in one strain from Morena, S929T in one strain from Ashoknagar and S943P observed in one strain of introductory index case from Gwalior district. Amino acid substitutions D614G was also observed in one of the fatal case, sequenced in this study.

### Phylogenetic analysis

**Figure 3:**
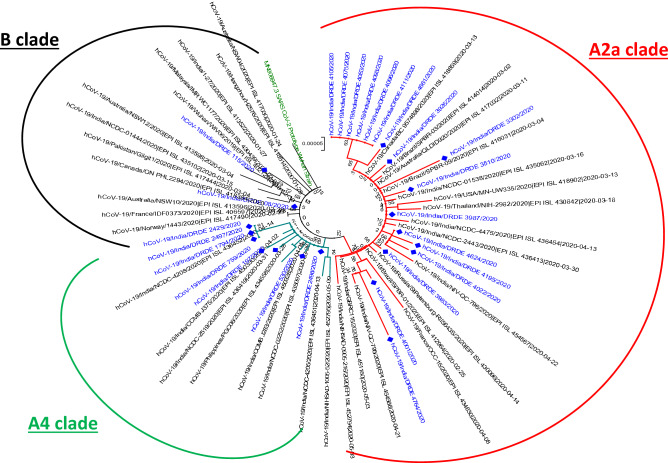
Phylogenetic analysis of Indian SARS-CoV-2 sequenced in this study. The virus sequence in this study were highlighted with blue color.

Whole genome based phylogenetic analysis was conducted for SARS-CoV-2 complete genomes (29.89 Kb, n = 37) retrieved from GISAID web interface and included the representatives from all location of globe e.g., Asia, Europe, Americas sampled between December 2019 to May 2020. The detailed molecular phylogenetic analysis revealed circulation of A1-A4 and B clades globally. Indian viruses were found belong to multiple evolutionary genetic clades (A2a, A3, A4 and B). However, the Indian viruses from Madhya Pradesh sequenced in this study belonged to three clades viz., A2a, A4 and B. The majority of virus were clustered in clade A2a (n = 17), whereas seven and two viruses were found belong to clade A4 and clade B respectively. The independent grouping with different family clusters of infection within region was also observed. The detailed phylogeny is depicted in Fig. 3. Another spike protein based phylogenetic tree was also constructed with 125 Indian circulating strains from different parts of India since lock down period which revealed continuous presence of D614G substitution in India (Supplementary Fig. S1).

## Discussion

The rapid transmission of SARS-CoV-2 and its expansion into geographical territories has led to devastating effect on global health and economy. India has been witnessing one of the worst burden of COVID-19 infections in the world. The early lockdown in India resulted in a pause in rise of new infections. However, following the mass movement of people across the nation during later phase of lockdown, there was an unprecedented expansion of pandemic in different states. Several reports have traced the evolutionary origins of SARS-CoV-2 from SARS CoV found in bats and pangolins^9^. The sudden emergence of SARS-CoV-2 in China is still not well understood. There is also a renewed interest to understand the evolutionary path being followed by SARS-CoV-2 to undertake effective control and mitigation measures^10^. In spite of large number of reported cases in India, the genomes of few Indian circulating viruses are available^11,12^. Further, there is a paucity of sequence data on the genetic evolution of SARS-CoV-2 circulating particularly in central India. This study summarizes the emergence and evolutionary patterns of SARS-CoV-2 from central Indian region particularly focusing the initial days of pandemic covering the lockdown and Unlock. These included the index cases, covering 10 different districts including whole Chambal region from Madhya Pradesh state during March to June 2020. The 26 whole genomes (29.89 kb) of SARS-CoV-2 viruses sequenced in this study have shown a number of mutations including non synonymous substitutions (responsible for change in protein) across the genome. These mutations spread across the genome and were reflected in ORF1ab, spike, matrix, envelope and nucleoprotein regions. High evolutionary activity was recorded in some regions particularly in the district of Morena, Shivpuri, Gwalior and Datia.

The first index case (A) was identified from district Shivpuri from a person with travel history to Hyderabad in southern India. This led to emergence of the virus in this region, prior to which only 10 cases were confirmed from the whole state. During the first lockdown period, another important index case (B) was reported from district Morena having travel history to Dubai. This index case led to emergence of SARS-CoV-2 from district Morena. The index cases C and D from district Sheopur and Gwalior respectively were traced to patients having travel history to Indore and United States respectively. Since these introductory events happened during stringent lockdown period, the infections were contained rapidly.

Following the lifting of restrictions, spread of infection was reported from different districts with a remarkable positivity in whole state. The highest number of case positivity (68%) was reported from district Morena in late lock down period, primarily due to reverse migration of workforce. The detailed analysis revealed multiple simultaneous introduction events with diverse geographical linkage. Phylogenetic analysis revealed a very distinct clade pattern of virus emergence and evolution in different districts. The introductory index cases (A and B) were found to belong to clade B, whereas, virus sequences from same district were found to be in clade A4/ A2a after a gap of one month during post lock down (Unlock).

Majority of Indian viruses sequenced in this study were found to belong to clade A2a (n = 17) as observed in many parts of the world^12–14^. This is a clade having travelers importation links to European countries particularly, Italy, UK, France. Another virus cluster (n = 7) belonged to clade A4 that comprised viral entries from Southeast Asia and central Asia. The initial index cases (A, B and C) were found to belong to a separate cluster (Clade B). The further expansions in viral emergence in these districts revealed a clear shift to worldwide circulating A2a and A4 clades. The independent grouping with different family clusters of infection within region was also observed. Interestingly, the A3 clade reported during this period was found absent in this part of India. The A3 clade was introduced to Ladakh union territory from Iran and Iraq during March 2020^12,14^. It was not further exported due to its geographical isolation and limited movement during lockdown period.

An important substitution (Spike: D614G) was identified in seventeen SARS-CoV-2 sequences in this study. D614G mutation in spike protein is an interesting substitution and has been reported with increased tally^15,16^. Structurally, this mutation is located in the S1 subunit that also contains the receptor binding domain (RBD). Although this is present outside the functional region, the proximity of D614G to S1 cleavage site implicates an important change in the local environment. In addition, the key mutation in spike protein (D614G) also involves loss of the charged group. This mutation that led to positively charged groups may cause more severe structural and functional effects. Recently, this mutation has been reported to make the virus more infectious. This mutation was further linked to higher number of cases and significant mortality rate in Europe^17,18^. This mutation was also recorded in one of the fatal cases sequenced in this study. In vitro studies also seem to support the hypothesis of increased transmissibility^19,20^. Another study suggested role of spike protein D614G mutation in increasing dominance and providing structural advantage to the furin cleavage domain^21^. However, experimental and in silico evidence suggests vaccines are unlikely to be affected by D614G mutation in SARS-CoV-2 spike protein^22^. This mutation also reported to increase transduction of multiple human cell types^23^. Presence of this mutation in majority of SARS-CoV-2 sequenced in this study indicates the circulation of this highly infectious virus variant in central part of India. Further, this mutation continued to persist in Indian strains till date.

Other notable significance is the P13L variant (C28311T) in the nucleocapsid (N) protein which is one of the most crucial structural components of the SARS-CoV. Further, this protein has also been proposed to be an efficient diagnostic molecule and a candidate vaccine for the SARS-CoV^22^. This amino acid substitution was recorded in four viral strains, including two index cases A and B from Shivpuri and Morena district respectively. The N protein mutation resides in the SR-rich region involved in viral capsid formation. Another important amino acid substitution P323L in RNA dependent RNA polymerase (RdRP) protein was recorded in 16 viral strains sequenced in this study. This amino acid substitution is also a specific mutation for clade A2a.

In summary, this is the first detailed genomic signature analysis of SARS-CoV-2 emergence and expansion in the Chambal region of Madhya Pradesh, central India focussing on the events of nation-wide lockdown and Unlock. This analysis also deciphered the initial introduction and later evolutionary events in Chambal region with respect to other parts of India during contemporary period. Continuous monitoring of genomic architecture of SARS-CoV-2 for possible mutations is crucial to identify virus variant responsible for increased transmission, virulence and drug resistance. Further, with the current focus on vaccination drive, rapid identification of escape mutants will be crucial for control of the pandemic waves.

## Methods

### COVID-19 laboratory screening

Nasopharyngeal/Nasal/Oropharyngeal swabs in viral transport medium (VTM) received from acute phase patients with defined symptoms, asymptomatic cases with contact history with positive patients/ travel history were processed for laboratory confirmation of SARS-CoV-2 at Defence Research and Development Establishment, (DRDE) Gwalior, M.P., India. These samples were referred for molecular diagnosis from 10 different districts health authorities of Madhya Pradesh, India during the period of March –May 2020 of COVID-19 pandemic. Samples were anonymized and de-identified so researchers were blind to the identity of the patients. Only secondary non-identifying data including age, sex, and travel history were provided. The experimental protocols of this study were approved by Institutional Biosafety Committee (IBSC) vide no IBSC/VIRO-04/20/PKD. Ethical approval and informed consent waiver for this study has also been granted vide no. VCH/VEC/June-2021/04 of Vidya Ethics Committee, Gwalior, India. All methods were performed in accordance with the relevant guidelines and regulations.

### Isolation of viral RNA

The samples were processed in a BSL-3 facility (High Containment Facility) following biosafety precautions at DRDE, Gwalior. The viral RNA was extracted from the referred samples using QIAamp viral RNA mini kit (Qiagen, Germany) as per manufacturer’s instructions. Briefly, 560 µl of lysis buffer was added to 140 µl of clinical specimen and incubated for 10 min at room temperature. The sample was passed through silica column followed by washing in 500 µl wash buffer and finally the viral RNA was eluted in 50 µl of elution buffer in a nuclease free tube and used as template in downstream molecular detection assay.

### Laboratory investigation of COVID-19

Presence of SARS-CoV-2 was investigated by performing SARS-CoV-2 screening (Envelope, Human Rnase P genes) and confirmatory assays targeting (RdRP, ORF 1ab, N genes) to ensure RNA quality during sample collection as per WHO protocol^24^. The Taqman qRT-PCR was performed with dual labeled probes with reporter dyes FAM/VIC (E/Rnase P- Screening Assay), FAM/VIC (RdRP/ORF- Confirmatory Assay), Cy5 (N gene- Confirmatory assay). The fluorescence signals were recorded in ABI 7500 Dx Real time PCR (ABI, USA).

### SARS-CoV-2 whole genome sequencing

The representative positive cases were selected on the basis of patients with travel history in the region (n = 6), patients with different age group from similar contact history (n = 10); introduction links from different areas to different families (n = 4) named as index case A(Shivpuri), index case B (Morena), index case C (Sheopur), index case D (Gwalior) COVID-19 positive death cases (n = 2) and index cases from different districts (n = 4). Based on above mentioned criteria, qRT-PCR positive cases (n = 26) were selected for whole genome sequencing. Briefly cDNA was synthesized using Superscript IV reverse transcriptase (NEB, USA) along with 23 mer oligo dT primer, 50 μM random hexamer and 10 mM dNTPs mix. The reaction was performed at 65 °C for 5 min and snap chilled on ice, Further reverse transcription was performed at 42 °C 50 min followed by 70 °C 10 min. The cDNA was stored at − 80 °C until further use.

### Amplification of whole genome by Oxford nanopore platform

SARS-CoV-2 whole genome sequencing was performed as per ARCTIC protocol by Josh Quick. Briefly, cDNA from samples were used as template for multiplex PCR to amplify SARS-CoV-2 genome and the amplified products were purified. Purified cDNA amplicons from each sample were barcoded individually and pooled at equimolar concentration to prepare nanopore library. The quality and quantity of the pooled library was assessed using standard methods and sequenced on GridION-X5 nanopore sequencer. To generate tiled PCR amplicons from the SARS-CoV-2 viral cDNA, primers were designed using primal scheme. These primers were pooled into three different primer sets named as pool 1, 2 and 3.

Briefly, multiplex PCR was performed with initial denaturation at 98 °C for 30 s followed by 25 cycles of denaturation at 98 °C for 15 s and annealing and extension at 65 °C for 5 min. Amplification from respective primers sets were confirmed by agarose gel electrophoresis. Raw data generated was subjected to analysis using ARCTIC protocol to generate consensus sequences for SARS-CoV-2 genome.

### Nanopore library preparation and sequencing

Amplified products obtained from multiplex PCR of pool 1, 2 and 3 were pooled and purified by using Ampure-XP beads. 50 ng amplified DNA from each samples was taken for library preparation using native barcoding kit and ligation kit from Oxford nanopore technology (ONT). Equimolar DNA from each sample was taken and end-repaired using NEBNext Ultra II End repair/dA-tailing module and cleaned with 0.4X Ampure XP Beads. Native barcode ligation was performed with NEBNext Ultra II Ligation module using Native Barcoding kit. All barcode ligated samples were pooled together and purified using 0.4X of AmPure beads. Further sequencing adapter ligation was performed using NEB next quick ligation module. Library mix was cleaned up using 0.4X AmPure beads and finally sequencing library was eluted in 15 μl of elution buffer and used for sequencing on SpotONflowcell in a 48 h sequencing protocol on GridION release 19.06.9. Nanopore raw reads (‘fast5’ format) were base called (‘fastq5’ format) and multiplexed using Guppy v3.2.2.

### Data analysis

Base-calling and demultiplexing of nanopore raw data was done using Guppy. The processed data was used for variant calling using Arctic pipeline and consensus generation using bam based method. The Arctic pipeline uses processed reads to align against the available SARS-CoV-2 reference genome. Further, variant calling and consensus sequence generation was done using Nanopolish. The good quality variants were used for annotation using snpEff tool.

### Genome analysis of SARS-CoV-2

The nucleotide sequences of representative SARS-CoV-2 were retrieved from Global Initiative on Sharing All Influenza Data (GISAID) and NCBI GenBank, edited and analysed employing EditSeq and MegAlign modules of lasergene5 software package (DNASTAR Inc, USA). The complete genome of 26 SARS-CoV-2 deciphered in this study was comparatively analysed with prototype SARS-CoV-2 isolated from Wuhan, China (GenBank Acc No MN908947). Multiple sequence alignment was carried out using MUSCLE alignment method in Bioedit software module. The amino acid substitutions of viruses sequenced in this study was compared to prototype SARS-CoV-2 Wuhan strain.

Phylogenetic analysis based on SARS-CoV-2 whole genomes were carried out with respect to globally diversified SARS-CoV-2 (n = 37) available at NCBI GenBank and GISAID data base from December 2019- May 2020 (Supplementary Table 2). The phylogenetic tree was constructed employing Neighbor Joining method with 1,000 replicates of bootstrap analysis with general time- reversible model with gamma distributed rates of variations among sites using Mega 5.03 software. Another S gene based phylogenetic tree was constructed by including 125 Indian SARS-CoV-2 strains from January 2020 to December 2020 employing the same Neighbor Joining method as described earlier.

## Supplementary Information

Supplementary Information.

## Data Availability

The data reported in this manuscript will be available to others without undue qualifications. The sequences reported in this study are deposited in global gene sequence repository for SARS-CoV-2 (GISAID, EpiCov) and corresponding accession numbers are provided in this manuscript.
