# Supplementary material for: Emergence and expansion of highly infectious spike protein D614G mutant SARS-CoV-2 in central India
Source: Sci Rep. 2021 Sep 13;11:18126. doi: 10.1038/s41598-021-95822-w (PMC8437943; doi:10.1038/s41598-021-95822-w)

SUPPLEMENTARY INFORMATION for

Emergence and expansion of highly infectious spike protein D614G mutant SARS -CoV-2 in central India

Shashi Sharma[1], Paban Kumar Dash[1*], Sushil K Sharma[1], Ambuj Srivastava[1], Jyoti S Kumar[1], B.S. Karothia[2], K T Chelvam[2], Sandip Singh[2], Abhay Gupta[2], Ram Govind Yadav[1], Ruchi Yadav[2], Greshma TS[2], Pramod Kushwah[2], Ravi Bhushan[2], D.P. Nagar[2], Manvendra Nandan[2], Subodh Kumar[2*], Duraipandian Thavaselvam[2], Devendra Kumar Dubey[2]

1- Virology Division, Defence Research Development Establishment

2- COVID-19 Diagnosis Task Force, Defence Research Development Establishment, Jhansi Road, Gwalior – 474002, India

Supplementary Files:

Supplementary Table 1: **Indian SARS -CoV-2 virus sequenced in this study.**

Virus Name	Type/GSAID ID	Gender	Collection Date	Location	Patient Age	Specimen Type
hCoV-19/India/DRDE 3510/2020	Betacoronavirus EPI_ISL_47688	Male	13-05-2020	Asia/India/M.P./Ashoknagar	29	Oro-pharyngeal swab
hCoV-19/India/DRDE 3926/2020	Betacoronavirus EPI_ISL_476884	Male	16-05-2020	Asia/India/M.P./Ashoknagar	27	Oro-pharyngeal swab
hCoV-19/India/DRDE 4078/2020	Betacoronavirus EPI_ISL_476885	Male	17-05-2020	Asia/India/M.P./Morena	09	Oro-pharyngeal swab
hCoV-19/India/DRDE 4083/2020	Betacoronavirus EPI_ISL_476886	Female	17-05-2020	Asia/India/M.P./Morena	11	Oro-pharyngeal swab
hCoV-19/India/DRDE 4093/2020	Betacoronavirus EPI_ISL_476887	Male	17-05-2020	Asia/India/M.P./Morena	06	Oro-pharyngeal swab
hCoV-19/India/DRDE 3983/2020	Betacoronavirus EPI_ISL_476888	Male	16-05-2020	Asia/India/M.P./Morena	20	Oro-pharyngeal swab
hCoV-19/India/DRDE 3987/2020	Betacoronavirus EPI_ISL_476889	Female	16-05-2020	Asia/India/M.P./Morena	35	Oro-pharyngeal swab
hCoV-19/India/DRDE 4022/2020	Betacoronavirus EPI_ISL_476890	Male	16-05-2020	Asia/India/M.P./Morena	29	Oro-pharyngeal swab
hCoV-19/India/DRDE 4096/2020	Betacoronavirus EPI_ISL_476891	Male	17-05-2020	Asia/India/M.P./Morena	38	Oro-pharyngeal swab
hCoV-19/India/DRDE 4105/2020	Betacoronavirus EPI_ISL_476892	Female	17-05-2020	Asia/India/M.P./Morena	35	Oro-pharyngeal swab
hCoV-19/India/DRDE 4111/2020	Betacoronavirus EPI_ISL_476893	Male	17-05-2020	Asia/India/M.P./Morena	31	Oro-pharyngeal swab
hCoV-19/India/DRDE	Betacoronavirus	Male	18-05-2020	Asia/India/M.P./Morena	39	Oro-pharyngea

4195/2020	EPI_ISL_476 894					l swab
hCoV-19/India/DRDE 4661/2020	Betacoronavirus EPI_ISL_476 895	Male	27-05-2020	Asia/India/M.P./Morena	24	Oro-pharyngeal swab
hCoV-19/India/DRDE 4624/2020	Betacoronavirus EPI_ISL_476 896	Male	25-05-2020	Asia/India/M.P./Datia	70	Oro-pharyngeal swab
hCoV-19/India/DRDE 3302/2020	Betacoronavirus EPI_ISL_476 854	Male	11-05-2020	Asia/India/M.P./Datia	12	Oro-pharyngeal swab
hCoV-19/India/DRDE 2498/2020	Betacoronavirus EPI_ISL_476 853	Male	04-05-2020	Asia/India/M.P./Jabalpur	32	Oro-pharyngeal swab
hCoV-19/India/DRDE 2497/2020	Betacoronavirus EPI_ISL_476 852	Male	04-05-2020	Asia/India/M.P.//Jabalpur	28	Oro-pharyngeal swab
hCoV-19/India/DRDE 2429/2020	Betacoronavirus EPI_ISL_476 850	Male	03-05-2020	Asia/India/M.P./Jabalpur	22	Oro-pharyngeal swab
hCoV-19/India/DRDE 1794/2020	Betacoronavirus EPI_ISL_476 849	Male	29-04-2020	Asia/India/M.P./Morena	23	Oro-pharyngeal swab
hCoV-19/India/DRDE 759/2020	Betacoronavirus EPI_ISL_476 848	Male	11-04-2020	Asia/India/M.P./Sheopur	37	Oro-pharyngeal swab
hCoV-19/India/DRDE 197/2020	Betacoronavirus EPI_ISL_476 846	Female	01-04-2020	Asia/India/M.P./Morena	14	Oro-pharyngeal swab
hCoV-19/India/DRDE 4001/2020	Betacoronavirus EPI_ISL_476 844	Male	12-05-2020	Asia/India/M.P./Gwalior	76	Oro-pharyngeal swab
hCoV-19/India/DRDE 4764/2020	Betacoronavirus EPI_ISL_476 842	Male	28-05-2020	Asia/India/M.P./Morena	35	Oro-pharyngeal swab
hCoV-19/India/DRDE 530/2020	Betacoronavirus EPI_ISL_476 840	Male	09-04-2020	Asia/India/M.P./Gwalior	55	Oro-pharyngeal swab
hCoV-19/India/DRDE 08/2020	Betacoronavirus EPI_ISL_476 022	Male	25-03-2020	Asia/India/M.P./Shivpuri	23	Oro-pharyngeal swab
hCoV-	Betacoronavi	Male	31-03-	Asia/India/M.P./Morena	38	Oro-

19/India/DRDE 115/2020	rus EPI_ISL_476 023		2020			pharyngea l swab

Supplementary Table 2: Details of Global SARS-CoV-2 virus sequences retrieved from GISAID web page.

Accession ID	Virus Name	Collection Date	Location
EPI_ISL_402129	hCoV-19/Wuhan/WIV06/2019	2019-12-30	China
EPI_ISL_406597	hCoV-19/France/IDF0373/2020	2020-01-23	Europe
EPI_ISL_412964	hCoV-19/Brazil/SPBR-01/2020	2020-02-25	Brazil
EPI_ISL_413522	hCoV-19/India/1-27/2020	2020-01-27	India
EPI_ISL_413596	hCoV-19/Australia/NSW10/2020	2020-02-28	Australia
EPI_ISL_413598	hCoV-19/Australia/NSW12/2020	2020-03-04	Australia
EPI_ISL_414014	hCoV-19/Brazil/SPBR-03/2020	2020-03-02	Brazil
EPI_ISL_416031	hCoV-19/Brazil/SPBR-09/2020	2020-03-04	Brazil
EPI_ISL_417030	hCoV-19/Australia/NSW04/2020	2020-01-24	Australia
EPI_ISL_417032	hCoV-19/Australia/QLDID920/2020	2020-03-11	Australia
EPI_ISL_417444	hCoV-19/Pakistan/Gilgit1/2020	2020-03-04	Asia
EPI_ISL_417490	hCoV-19/Norway/1443/2020	2020-02-27	Europe
EPI_ISL_418384	hCoV-19/Canada/ON_PHL2294/2020	-	North America
EPI_ISL_418514	hCoV-19/Hangzhou/HZ576/2020	2020-01-25	China
EPI_ISL_418859	hCoV-19/Canada/BC_9574898/2020	2020-03-13	North America
EPI_ISL_418902	hCoV-19/USA/MN-UW335/2020	2020-03-13	USA
EPI_ISL_430096	hCoV-19/Russia/StPetersburg-RII5643S/2020	2020-04-14	Russia
EPI_ISL_430439	hCoV-19/Malaysia/IMR_WC1177/2020	2020-03-05	Asia

EPI_ISL_430842	hCoV-19/Thailand/NIH-2982/2020	2020-03-18	Asia
EPI_ISL_434558	hCoV-19/Philippines/PGC06/2020	2020-03-28	Asia
EPI_ISL_434630	hCoV-19/France/OCC-15/2020	2020-04-08	Europe
EPI_ISL_435062	hCoV-19/India/NCDC-01538/2020	2020-03-16	India
EPI_ISL_435097	hCoV-19/India/NCDC-02252/2020	2020-03-28	India
EPI_ISL_435102	hCoV-19/India/NCDC-01444/2020	2020-03-15	India
EPI_ISL_436413	hCoV-19/India/NCDC-2443/2020	2020-03-30	India
EPI_ISL_436419	hCoV-19/India/NCDC-2519/2020	2020-03-31	India
EPI_ISL_436451	hCoV-19/India/NCDC-4205/2020	2020-04-13	India
EPI_ISL_436452	hCoV-19/India/NCDC-4208/2020	2020-04-14	India
EPI_ISL_436454	hCoV-19/India/NCDC-4475/2020	2020-04-13	India
EPI_ISL_450328	hCoV-19/India/CCMB_J283/2020	2020-04-01	India
EPI_ISL_450330	hCoV-19/India/CCMB_J375/2020	2020-04-02	India
EPI_ISL_451160	hCoV-19/India/GBRC115/2020	2020-05-03	India
EPI_ISL_452794	hCoV-19/India/NIHSAD-0905-216/2020	2020-05-08	India
EPI_ISL_452795	hCoV-19/India/NIHSAD-1005-52/2020	2020-05-09	India
EPI_ISL_454566	hCoV-19/India/NIV-QC-795/2020	2020-04-21	India
EPI_ISL_454567	hCoV-19/India/NIV-QC-	2020-04-22	India

	796/2020		
MN908947	Wuhan-Hu-1	Dec-2019	China

Supplementary Fig S1: Phylogenetic analysis of Indian SARS- CoV-2 spike gene with respect to other Indian viruses circulating till date. The virus sequence in this study were highlighted with blue color.

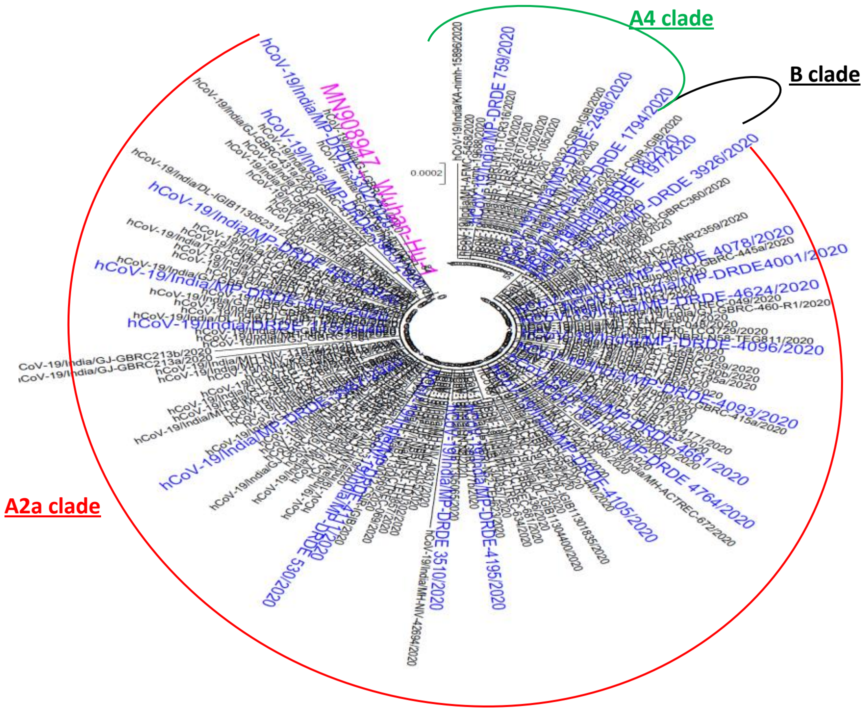

Supplement: Supplementary file 1 — Supplementary Information. [file 41598_2021_95822_MOESM1_ESM.pdf]
